# Angioedema as a Rare Presentation of Systemic Lupus Erythematosus

**DOI:** 10.7759/cureus.31763

**Published:** 2022-11-21

**Authors:** Faria L Sami, Hania L Sami, Hamza Tahir, Joseph Guan

**Affiliations:** 1 Internal Medicine, John H. Stroger, Jr. Hospital of Cook County, Chicago, USA; 2 Anatomy, Physiology, Biochemistry, Shalamar Medical And Dental College, Lahore, PAK; 3 Internal Medicine, Allama Iqbal Medical College, Lahore, PAK

**Keywords:** internal medicine and rheumatology, bradykinin mediated angioedema, lupus flare, angioedema, systemic lupus erythromatosus

## Abstract

Angioedema (AE) is an immune-mediated tissue swelling that can be life-threatening if it compromises the airway. This makes prompt diagnosis and management of the condition excruciatingly important. It can be hereditary or associated with infections, malignancies, and autoimmune diseases. There have been reported cases in the literature where Systemic lupus erythematosus (SLE) patients developed acquired angioedema raising suspicion of a possible association between the two conditions.

We describe a case of a patient with no known medical issues, presenting with acute onset of her first episode of angioedema with airway compromise. Because of the rarity of awareness of the possible association of our conditions of interest, there was an inevitable delay in diagnosis and the patient was eventually diagnosed to have SLE and associated acquired angioedema as its first presentation.

This case report highlights the importance of maintaining high suspicion for SLE in patients with an isolated first episode of AE and discusses mechanisms involved in the disease process to shed light on available treatment modalities.

## Introduction

Systemic lupus erythematosus (SLE) is a systemic inflammatory condition with autoimmune pathophysiology, characteristic of autoantibodies causing end-organ damage [1]. The clinical presentation is widely variable including cardiovascular emergencies (pericarditis, cardiac tamponade, myocardial infarction, thrombosis), neurological (stroke), pulmonary (pulmonary hemorrhage/ edema), and even renal (lupus nephritis) [2]. Since mortality in SLE patients is approximately five times higher than in the general population, it is very important to timely recognize different presentations of lupus patients, and to initiate the appropriate treatment without delay. 

Angioedema (AE) can be a life-threatening immune-mediated inflammation of skin/subcutaneous tissue risking airway compromise. Also known as angioneurotic edema, AE is associated with 15-33% of SLE patients [3]. However, it is important to establish it as the first clinical presentation of SLE for timely diagnosis. It is to emphasize a prompt systematic approach for management to reduce mortality and morbidity from AE in SLE patients. 

We present a rare case of facial angioedema as the first presentation of SLE, discussing the barriers in diagnosis and delayed treatment due to the rarity of the presentation.

## Case presentation

A 39-year-old woman with no significant past medical history presented to the emergency department (ED) with the chief complaint of acute onset of bilateral submandibular swelling for the past day. However, her symptoms worsened to the point of progressive dysphagia, increasing oral secretions, and oral pain which prompted her to get medical attention. She denied having any fever or difficulty breathing. A review of systems was otherwise unremarkable. She remained afebrile and hemodynamically stable on arrival at the ED. The patient’s medication list was unremarkable for any triggering medications including angiotensin-converting enzyme inhibitors (ACEi) or angiotensin receptor blockers (ARBs). On physical examination, there was notable bilateral submandibular and submental swelling and pink-colored depigmented scaly plaques with hyperpigmented borders on the face and forehead. There were no signs of tongue or lip swelling initially. 

**Figure 1:**
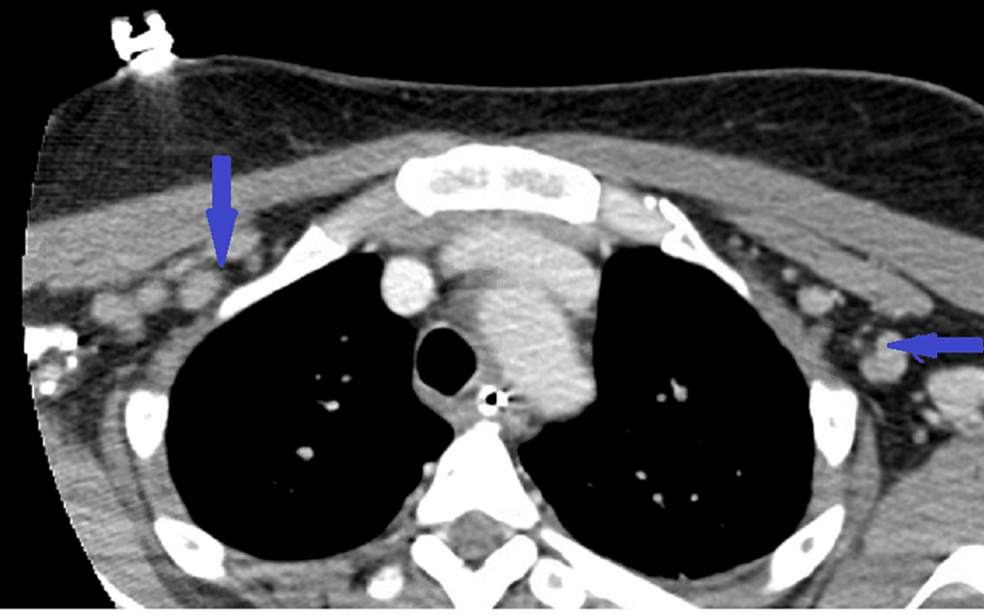
Bilateral axillary and pre-pectoral lymphadenopathy was noted on CT chest (blue).

**Figure 2:**
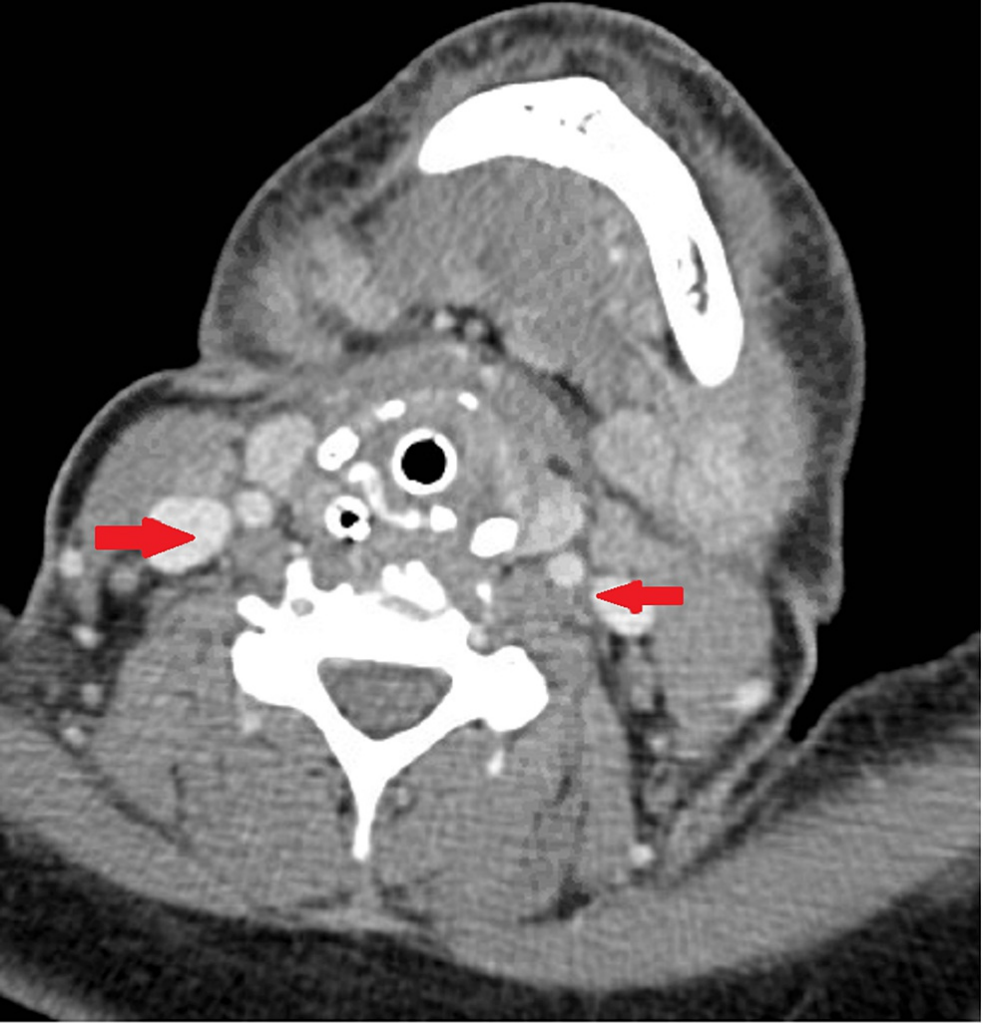
Bilateral suprahyoid and infrahyoid lymphadenoapthy (red) with inflammatory changes involving the soft tissues of larynx/pharynx are seen.

Initial laboratory findings revealed mild leukocytosis (WBC 12.4x10^3/L), elevated erythrocyte sedimentation rate (ESR) of 97 mm/hr, microcytic anemia (Hb 9.5 mg/dl, MCV 70.1), and thrombocytosis (platelet count 421x10^3/L. Reticulocyte count was 1.4 %, Iron 17 ug/dL, Total Iron Binding Capacity 216 ug/dL, % saturation 7.9 %, Ferritin 9.37 ng/mL, Vitamin B 12 295.9 pg/mL and folate 6.57 ng/mL. Contrast computed tomography (CT) of the neck and soft tissue showed multilevel bilateral cervical and axillary/pre-pectoral lymphadenopathy (LAD) (Figures 1, 2), with the largest measuring approximately 2 cm. No evidence of airway obstruction was visualized. At the time of presentation, she was suspected to have Ludwig’s Angina in the setting of bilateral submandibular swelling, increased oral secretions, dysphagia, and acuity of progression.

She was started on empiric treatment for Ludwig’s angina after an ENT evaluation with levofloxacin, and metronidazole. Intravenous (IV) diphenhydramine and IV dexamethasone were also administered for a presumed allergic reaction and to protect against airway edema. Despite initiating treatment, her symptoms continued to progress in the ED where she began having difficulty tolerating secretions, tongue swelling, and airway compromise. She was intubated for airway protection and was taken emergently to the operating room by oral maxillofacial surgery service for incision and drainage (I&D) and excisional lymph node biopsy evaluation for possible lymphoproliferative disease in the setting of diffuse LAD. However, I&D did not express the obvious purulence that would be expected for an abscess. Afterward, she was shifted to the surgical intensive care unit (SICU) for post-procedural management.

With only mild leukocytosis, lack of systemic signs of infection, and no identifiable source of infection, alternative etiologies of swelling were considered at this point including autoimmune angioedema. C1q and C1 esterase inhibitor levels were sent and reported to be within normal limits. Allergy service was consulted for further workup of angioedema, but given the patient’s age of presentation, normal level of C1q, and C1 esterase inhibitor, there was lower suspicion for hereditary and acquired angioedema.

The dermatology service was consulted to weigh in on whether the patient’s facial rash was related to her presentation. At this time, it was believed that the rash was consistent with discoid lupus erythematosus (DLE), but was unlikely to explain her clinical presentation. The possible diagnosis of systemic lupus erythematosus (SLE) came to light and prompted a further workup. Rheumatologic workup was positive for anti-nucleic acid antibody (ANA) (titer > 1:160), anti-Smith (anti-Sm) antibody (> 8 IU/L), anti-ribonucleotide reductase antibody (anti-RNP) (> 8), anti-SSA antibody(> 8 IU/L)), and low C3 (82 mg/dL) and C4 (14 mg/dL) complement proteins (lower limit normal was 88 mg/dL and 16 mg/dL, respectively). 

By this time, the pathology report from the excisional lymph node biopsy had revealed nodal sclerosis and large bands of fibrosis consistent with connective tissue disorder. Rheumatology was consulted and given the severity of the patient’s clinical status, presentation, and serological workup, treatment for SLE with angioedema was initiated with IV prednisone 40 mg daily and cetirizine 20 mg twice daily. After the initiation of this treatment regimen, her clinical course immediately began to improve with the eventual resolution of angioedema on subsequent CT scans.

In the setting of an improving disease course, the diagnosis of SLE with severe angioedema became more definitive. After 13 days post-I&D, she was extubated and was discharged on a tapering prednisone dose, azathioprine 50 mg daily, and hydroxychloroquine 200 mg daily for maintenance management of SLE to follow up in the clinic.

On her subsequent rheumatology clinic follow-ups, she was doing well and angioedema had not recurred. However, she reported developing oral discoid lesions (which were attributed to azathioprine use) and new Raynaud’s phenomenon in her hands and feet. She was advised to continue the prednisone taper, azathioprine, and hydroxychloroquine. Unfortunately, she was lost to follow-up after her initial outpatient rheumatology post-hospital evaluation and her current clinical picture is unclear.

## Discussion

Angioedema (AE), also known as angioneurotic edema, is the acute onset of immune-mediated skin tissue edema involving the subcutaneous layer mostly commonly in the periorbital region or lips [4]. It can be either hereditary (HAE) or acquired which is further characterized depending on distinct pathophysiology [5]. There are two common types of hereditary angioedema depending on C1-esterase inhibitor mutations affecting either secretion or functionality of the protein [6]. A third type is a familial form with normal C1 esterase inhibitor (C1-INH) titers and function [7]. 

Acquired angioedema (AAE) can be associated with lymphoproliferative diseases such as lymphoma, monoclonal gammopathy of uncertain significance, neoplasias, infections, and, more recently coming to light, systemic lupus erythematosus (SLE) [8].

Two main mechanisms are known for AAE. Type 1 involves accelerated catabolism of C1 esterase inhibitor and type 2 is characterized by the presence of an autoantibody against the enzyme [9]. 

Patients with concomitant SLE and Angioedema are relatively younger and females of African-Americans descent. The definitive pathophysiology of AAE in SLE is unclear at this time. Hereditary AE has been reported in SLE patients, but there is an increasing concern about AAE in SLE as well now. SLE patients have demonstrated a significantly high number of acetylated modifications of C1-inhibitor (C1-INH) and high autoantibody titers for C1-INH [10]. While another study suggests that C1-INH levels can be normal in SLE individuals, but significantly less reactive to C1s and C1r with no identified autoantibodies or mutations. This study on 8 patients raised the possibility of distinct pathophysiology for SLE AAE not previously known [11]. In summary, lymphoproliferative disease (LPD) can be associated with catabolizing C1-INH or with antibodies that inactivate C1-INH. Our case describes a rare scenario without LPD or anti-C1-INH antibodies in SLE patients coming with facial angioedema [12]. 

SLE and AAE patients may have other comorbid conditions but lymphoma should be ruled out in most high-risk individuals with AAE. Treatment and management are urgently indicated for airway protection and to avoid fatalities. There may be serological evidence of C1-INH deficiency as well as low C1q, C3, and C4, but also evidence of increased C1-INH catabolism in the setting of no response in complement levels with CI-INH infusion [13]. Autoimmune workup for SLE is remarkable and inflammatory serum markers such as ESR, and CRP will be elevated in these patients. Concomitant treatment of SLE with steroids, cyclophosphamide, hydroxychloroquine, etc in AAE-diagnosed patients has shown complete resolution of symptoms which emphasizes that the association is not likely by chance [12, 13]. Another new treatment option is ecallantide for refractory angioedema in SLE patients, with normal C1-INH levels. 

We present a case of life-threatening angioedema as the first presentation of SLE, which was misdiagnosed resulting in prolonged ICU stay and intubation. Due to low concern of AAE as a presentation of SLE, the patient was treated for multiple other etiologies with futility before initiation of lupus treatment with pulse steroids and azathioprine, which showed continued marked improvement thereafter.

HAE may be seen in up to 2 % of SLE patients. On the other hand, acquired AE in lupus is rare and the prevalence of SLE in AE is expected to be less than 1% [14]. Therefore, for diagnostic purposes, there should be suspicion of non-allergic causes as well. C1-INH with complement levels can aid in diagnosis. If both are within normal range, diagnosis of C1-INH deficiency can be excluded, however, functional deficiency can still not be ruled out.

Immunosuppressive therapy results in the normalization of C3, C4, and C1-INH levels along with the resolution of angioedema [15]. Lupus patients with AAE may have an increased predilection towards central nervous system (CNS) involvement due to the increased vascular permeability in the CNS secondary to bradykinin and psychosis is usually a predominant feature. Among AE-related comorbidities in patients with SLE, the atopic disorder, Leukocytoclastic vasculitis, infections, and eosinophilia are common.

There’s a “third type” of C1-INH-AAE in clinically silent SLE patients with low C1-INH antigenic and functional levels along with hypocomplementemia, both of which normalized with a resolution of AE after immunosuppressive therapy. Research has also shown that lupus patients are at higher odds of having AE in an inpatient setting, including severe AE as the principal reason for inpatient admission after adjusting for major comorbidities and medication adverse effects [16]. If there is C1-INH deficiency, C1q levels are found to be decreased in about 70% of patients with AAE, compared with HAE [17].

If the cause is determined to be C1-INH deficiency due to increased breakdown, both Danazol and Stanozolol (synthetic steroids) can be tried for treatment, although there have been reports of the paradoxical flare of lupus-like disease when both HAE, as well as non-C1 INH dependent angioedema, were treated with danazol. Rituximab has also demonstrated success in treatment-resistant acquired angioedema due to a deficiency of C1-INH inhibitor in a small pool of patients [18]. 

Most patients are treated with high-dose steroids - both for angioedema and active lupus, though lupus activity may be suppressed at the time of presentation with AAE. Steroids work to reduce angioedema swelling caused by non-histaminergic mechanisms, with minimal adverse effects [19]. In severe emergency cases, C1-INH can also be administered, as well as a fresh frozen plasma or solvent/detergent-treated plasma [20].

## Conclusions

Well-timed diagnosis and management of AE that is the first presentation of SLE can help reduce morbidity and mortality in our patients. It is important to realize the mechanism that is more commonly C1-INH dependent to be able to provide the most accurate treatment. High suspicion should be maintained for SLE among possible etiologies when patients present with isolated, first episode of AE. Treatment of SLE in AAE patients with high dose steroids, aids in therapy, as well as C1-INH concentrate, rituximab and fresh frozen plasma as part of general management of life-threatening AE.
